# *Aspergillus flavus* as a Model System to Test the Biological Activity of Botanicals: An Example on *Citrullus colocynthis* L. Schrad. Organic Extracts

**DOI:** 10.3390/toxins11050286

**Published:** 2019-05-22

**Authors:** Francesca Degola, Belsem Marzouk, Antonella Gori, Cecilia Brunetti, Lucia Dramis, Stefania Gelati, Annamaria Buschini, Francesco M. Restivo

**Affiliations:** 1 Department of Chemistry, Life Sciences and Environmental Sustainability, University of Parma, Parco Area delle Scienze 11/A, 43124 Parma, Italy; lucia.dramis@unipr.it (L.D.); annamaria.buschini@unipr.it (A.B.); restivo@unipr.it (F.M.R.); 2 Laboratory of Chemical, Galenic and Pharmacological Development of Drugs, Faculty of Pharmacy of Monastir, University of Monastir, 5000 Monastir, Tunisia; belsemmarzouk@yahoo.fr; 3 Tree and Timber Institute (IVALSA), National Research Council of Italy (CNR), Via Madonna del Piano 10, 50019 Sesto Fiorentino, Firenze, Italy; antonella.gori@unifi.it (A.G.); cecilia.brunetti@unifi.it (C.B.); 4 Department of Agriculture, Environment, Food and Forestry (DAGRI), University of Florence, Piazzale delle Cascine 18, 50144 Firenze, Italy; 5 Department of Packaging, Experimental Station for the Food Preserving Industry (SSICA), Viale Tanara 31/A, 43121 Parma, Italy; stefania.gelati@ssica.it; 6 Center for Molecular and Translational Oncology, Parco Area delle Scienze, 43124 Parma, Italy

**Keywords:** antimycotoxigenic activity, *Citrullus colocynthis*, *Aspergillus flavus*, model system, HPLC-MS/MS

## Abstract

*Citrullus colocynthis* L. Schrader is an annual plant belonging to the Cucurbitaceae family, widely distributed in the desert areas of the Mediterranean basin. Many pharmacological properties (anti-inflammatory, anti-diabetic, analgesic, anti-epileptic) are ascribed to different organs of this plant; extracts and derivatives of *C. colocynthis* are used in folk Berber medicine for the treatment of numerous diseases—such as rheumatism arthritis, hypertension bronchitis, mastitis, and even cancer. Clinical studies aimed at confirming the chemical and biological bases of pharmacological activity assigned to many plant/herb extracts used in folk medicine often rely on results obtained from laboratory preliminary tests. We investigated the biological activity of some *C. colocynthis* stem, leaf, and root extracts on the mycotoxigenic and phytopathogenic fungus *Aspergillus flavus*, testing a possible correlation between the inhibitory effect on aflatoxin biosynthesis, the phytochemical composition of extracts, and their in vitro antioxidant capacities.

## 1. Introduction

Oxidation is considered an underlying mechanism in the incidence of chronic diseases: Reactive oxygen species (ROS) such as superoxide anions, hydroxyl radicals, and hydrogen peroxide are cytotoxic, leading to tissue injuries. As in a “domino effect”, oxidative stress resulting from the imbalance between the generation of reactive oxygen species and endogenous antioxidant systems induces inadvertent enzyme activation and consequent oxidative damage to cellular systems [1]. It is widely reported that cellular oxidative damage is responsible for numerous disorders, such as cardiovascular [2], Alzheimer’s [3], and Parkinson’s disease [4]—as well as ulcerative colitis [5], atherosclerosis [6], and cancer [7]. A key defense mechanism against radical mediated toxicity is represented by antioxidants, which protect cells from the damage caused by free radicals [8]. Consequently, during the last thirty years, several antioxidant-based formulations for the prevention and treatment of complex diseases have been developed [9,10,11]. Among these antioxidant formulations, a high number of plant-derived drugs, widely used for ethnopharmaceutical preparations, have been applied as “natural” principles included in modern medical applications [12,13]. Accordingly, interest in botanicals as a source of bioactive compounds has increased worldwide, and the finding of new biologically remarkable natural compounds affects not only the pharmaceutical field, but the nutraceutical and cosmeceutical fields too [14,15,16,17]. Clinical studies aimed at confirming the scientific bases (chemical and biological) of pharmacological activity of many plants used in folk medicine often rely on the results obtained from preliminary laboratory tests. For example, the antioxidant properties of plant extracts are typically evaluated through in vitro analyses, such as DPPH (2,2-diphenyl-1-picryl-hydrazyl-hydrate) and ABTS [2,2′-azinobis-(3-ethylbenzothiazoline-6-sulfonate)] assays [18], and to date, the number of in vitro studies far exceeds the number of in vivo studies, remaining the most cost effective and predominant type of research investigations performed. In fact, factors including costs, interspecific differences that preclude the adequate predictive value of the experiments, feasibility of testing procedures, and ethical concerns generally limit the utilization of animal models and human subjects for this kind of research [19]. The possibility of using high-throughput small-scale in vivo or ex vivo model systems to predict a possible antioxidant biological activity of plant extracts before their medical application is therefore desirable. Eukaryotic microorganisms, though few, have sometimes been selected for this purpose: For example, the yeast *Saccharomyces cerevisiae* has been employed to determine the antioxidant activity of different berry juices, which reportedly contain high amounts of phenolics [20].

*Aspergillus flavus*, a saprophytic plant pathogen, is the predominant species producing aflatoxins (AFs). Among multiple events that contribute to aflatoxin production, those involving ROS accumulation—such as during morphological and metabolic transitions—and the establishment of an oxidative intracellular environment seem to possess a key role in triggering AF biosynthesis. This correlation has been supported by the identification of at least one transcription factor, which responds to the cellular oxidative stress by activating a series of enzymes responsible for the scavenging of cytoplasmic ROS excess [21,22,23]. Conversely, many compounds with antioxidant properties (such as ascorbate, eugenol, ethylene, methyl jasmonate, and α-lipoic acid) showed to exert an inhibition/containment effect on AF biosynthesis [24,25,26,27], mainly through the stimulation of catalase, superoxide dismutase, and glutathione peroxidase activity [28]. An inhibitory effect on AF biosynthetic pathway and *A. flavus* growth has been recently reported for a wide number of botanicals and essential oils [29,30,31]. Since the response of aflatoxin metabolism to redox balance alterations is well known, as they are considered a sort of “defense molecule” synthesized to cope with an excess of ROS in the late phase of growth [22,23], we proposed this airborne microorganism as a model system to screen the antioxidant potential of plant extracts.

Here we analyzed the effect of organic extracts of *Citrullus colocynthis* L. Schrader, an annual plant belonging to the Cucurbitaceae family which grows in arid and semi-arid regions, on AF biosynthesis and *A. flavus* growth. Native to tropical Asia and Africa, *C. colocynthis* is is now widely distributed in the desert areas of the Mediterranean basin (in Italy the only known population is located in the Aeolian island of Vulcano). Many pharmacological properties (anti-inflammatory, anti-diabetic, analgesic, anti-epileptic) are ascribed to different organs of this plant [32,33,34,35]: Extracts and derivatives of *C. colocynthis* are used in folk Berber medicine for the treatment of numerous diseases; the root is used for arthritic pain, breast inflammation, ophthalmia, and uterine pain; and the leaves are used for treatment of cough, many tumors, and as a cholagogue [36]. Recently, antifungal and antibacterial activities of organic extracts from leaves and seeds were reported [37,38,39]. However, despite various studies on the medical use of *C. colocynthis* derivatives, information about its antimycotoxigenic potential are still scarce. The aim of this work is to evaluate the antiaflatoxigenic effect of organic extracts of *C. colocynthis* stem, leaf, and root through the use of *A. flavus* as a model system, comparing their effect on the basis of phytochemical composition.

## 2. Results and Discussion

### 2.1. Phytochemical Characterization of C. colocynthis Extracts

**Figure 1:**
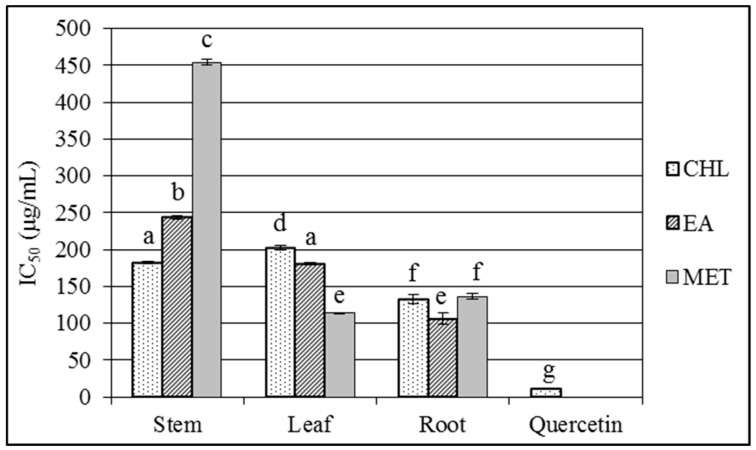
DPPH scavenging activity of *Citrullus colocynthis* root, leaf, and stem extracts. Data are means of three replicates ± S.D. Same letters indicate absence of statistically significant differences (*p* < 0.05).

Several studies reported a high in vitro antioxidant potential of organic *C. colocynthis* extracts obtained from various tissues, due to their polyphenolic composition [40,41]. The antioxidant capacity of root, stem, and leaf extracts and phenolic content were then measured according to the DPPH and Folin-Ciocalteu’s methods, respectively. As a general consideration, it should be noted that stem and leaf extracts showed a wider range of antioxidant activity, depending on the extraction solvent, than root extracts (Figure 1). The highest antioxidant capacity was determined in the methanol (MET) leaf extract and ethyl acetate (EA) root extracts, followed by chloroform (CHL) and methanol root extracts. On the contrary, MET stem extracts showed the lowest activity (Figure 1).

A detailed phytochemical characterization of the leaves, stems, and roots of *C. colocynthis* revealed the presence of different classes of metabolites—such as coumarins, hydroxycinnamic acid derivatives, flavan-3-ols glycosides, flavone glycosides, and tetracyclic triterpenes (Table 1)—as according to previous investigations [42,43,44]. These compounds, which have been indicated as responsible for antifungal activity against *Aspergillus* strains [38], were abundant in the analyzed extracts (Table 1). Esculetin (1), p-coumaric acid derivatives (2, 5), orientin (3), vitexin (6), apigenin derivatives (4, 7, 8, 9, 12, 17), and epicatechingallate (18) were identified according to their MS fragmentation pattern and absorption spectra (Table 1). The remaining identified peaks corresponded to different flavone derivatives (10, 11), and cucurbitacin derivatives (13, 14, 15, 16) were elucidated by their molecular weight obtained by MS analysis, their UV spectra, and by comparing experimental data with respective literature data (Table 1) [45,46,47]. In particular, cucurbitacin derivatives and colocynthoside B did not furnish any MS fragment as previously reported by Chawech et al. [44]. In order to exclude that these compounds were artefacts due to solvent extraction, they were also compared with MS and UV spectra of authentic standards of cucurbitacin E and I. Compound **19** (Table 1) was tentatively identified as colocynthoside B on the basis of its molecular weight, its UV spectrum, and the comparison with literature data [48]. Marked differences were observed among the tissues. However, in all three extracts, the leaf contained the highest variety of phenolics, as well as the higher content of secondary metabolite, except for compound **12** (Table 1), which was more abundant in the ethyl acetate extract of the stem. Overall, our results showed that ethyl acetate was the most efficient solvent to extract phenolic constituents in all tissues, apart from orientin, coumaric flavone derivative, two apigenin hexosides (compounds **7** and **17**), epicatechin gallate, vitexin, and compound **5**—which were more concentrated in methanol extracts. On the contrary, chloroform was efficient to extract cucurbitacin I derivatives (compounds **13**, **14**, **16**) and colocynthoside B. The most abundant cucurbitacin derivative (compound **13**) resulted in extraction to the same extent as in ethylacetate and chloroform. The presence of cucurbitacins is relevant to the bitterness and toxicity of the plant, but it also has some biological effects—such as anti-inflammatory, purgative, and anti-cancer activities [49].

### 2.2. Antifungal and Anti-Aflatoxigenic Activity

The prediction of the biological activity of natural extracts may often be difficult due to the variation in their chemical constituents, that in turn depend on the growth stages of the plants, and/or their geographic origin [50]. On the other hand, screening plant crude extracts can simplify the discovery of new and promising bioactives, and allows a further, more specific identification of the chemical compounds responsible for the observed activity. We performed a preliminary assay to evaluate the effect of the different *C. colocynthis* extracts on *A. flavus* growth, intended as daily radial increase of fungal colonies diameter. Concentration of 500 µg/mL was tested for each extract. As reported in Table 2, none of them resulted in a significant reduction of radial mycelium growth, suggesting that the composition of extracts did not possess appreciable antimicrobial activity against the fungus.

These preliminary results led to the exclusion of any antifungal or fungistatic potential of extracts on the mycelium long-term growth. However, various studies have reported that several compounds, both synthetic and natural, are effective in lowering AF production without apparently interfering with the fungal development [51,52]. At present, these compounds are a promising tool for uncovering the regulatory mechanisms triggering the mycotoxigenic metabolism, one of the main targets for mycotoxin diffusion/contamination control strategies [53,54].

**Figure 2:**
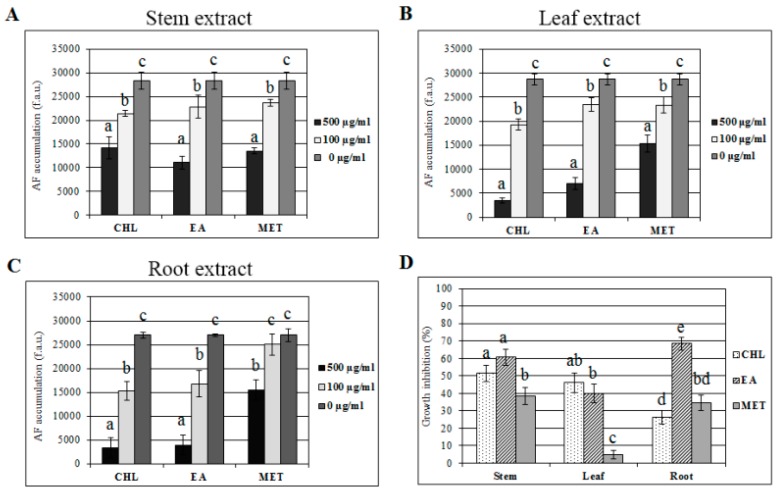
Activity on toxin accumulation and mycelium growth. Aflatoxin accumulation (reported as fluorescence arbitrary units) in *A. flavus* six-days after CCM cultures treated with stem (**A**), leaf (**B**), and root (**C**) extracts. (**D**) Early mycelium growth inhibition of *A. flavus* conidia treated with 500 µg/mL extracts, measured 48 h after inoculum by optical density increasing, and expressed as percentage in respect to control. Error bars refer to mean values of four replicates ± S.D.

To assess and compare the efficacy of different organic extracts of *C. colocynthis* tissues on total AF biosynthesis, conidia of *A. flavus* were inoculated in clarified coconut medium (CCM) and the fluorescence-based microplate procedure was used [27]. Leaf, stem, and root organic extracts were tested at increasing concentrations (data not shown); the lower and the higher concentrations (100 and 500 μg/mL, respectively) are reported in Figure 2. Chloroform (CHL), ethyl acetate (EA), and methanol (MET) extracts were administrated to aflatoxigenic *A. flavus* cultures. After six days of incubation, aflatoxin accumulation showed a dose-dependent alteration in response to extract exposure: The lowest dose (100 μg/mL) was less effective in limiting the amount of toxins in the culture, while the effect increased when extracts were added at the concentration of 500 μg/mL. Among tissues, leaf and root extracts had the highest levels of aflatoxin inhibition (Figure 2B,C), exceeding 80% inhibition in both CHL extracts. In addition, root extracts were able to lower the aflatoxin concentration by up to 12% in the most effective (CHL), and around 45% in the case of the least effective (MET; Figure 2B).

A similar correlation between solvent and aflatoxin inhibition rate was observed for the highest concentration of EA leaf and root extracts in eculetin, p-coumaric acid, apigenin, and cucurbitacin derivatives (Table 1). Interestingly, the high activity of the CHL extract could be related to cucurbitacin derivatives and colocynthoside B. In particular, colocynthoside B was detected only in the chloroform extracts and may be responsible of the observed aflatoxin inhibition (Figure 2). Prevention of AF accumulation has, for a long time now, been associated with the action of molecules and/or conditions that interfere with fungal growth [24,29,30]; however, during the last decades, several other substances have been shown to be effective in completely blocking the biosynthesis of mycotoxins without affecting mycelium development [52,55]. Thus, the correlation between aflatoxin metabolism and *A. flavus* growth should be considered under different perspectives of fungal development: Colonies may have the same radius, but vary significantly in hyphal density and, therefore, biomass. In fact, hyphae branching, which is necessary for an efficient colonization and utilization of the substrate, responds to nutrient gradients, growing away from areas staled by metabolic by-products of existing hyphae. However, colony radial growth is not influenced by the concentration of nutrients, since existing hyphal tips at the colony margin, which determine the colony diameter, have priority over all other hyphal tips (i.e., the branches). For this reason, the evaluation of colony radial growth as a unique parameter for the assessment of any antifungal effect could be misleading about possible fungistatic activities of tested compound/mixture, or might disguise an early stadium effect. As reported in Figure 2D, when *C. colocynthis* extracts were administrated to *A. flavus* conidia in YES liquid cultures at the higher concentration (500 µg/mL), early mycelium development was delayed by the majority of extracts. The exerted effect depended either on the tissue or solvent: For example, while CHL and EA extracts from the stem and leaf did not significantly differ in their inhibitory effect (50 vs. 60% and 45 vs. 50%, respectively), EA root extract proved to be more highly effective against the initial development of mycelium than CHL extract from the same tissue (70 vs. 25%).

### 2.3. Aflatoxin-Modulating Activity

**Figure 3:**
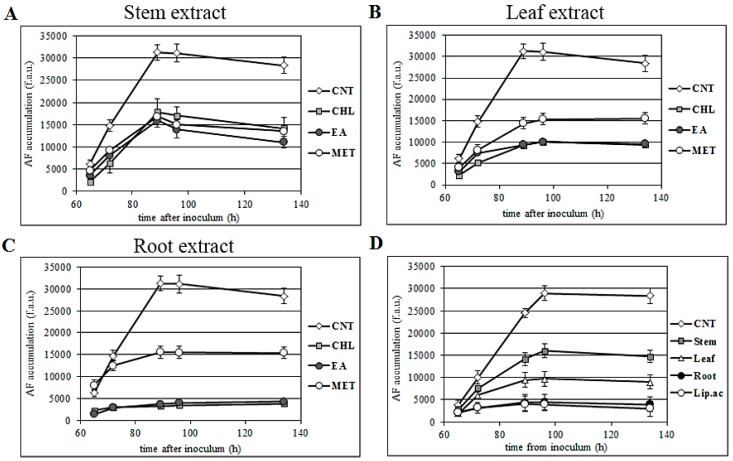
Aflatoxin time-course accumulation. Effect of stem (**A**), leaf (**B**), and root (**C**) extracts on AF time-course accumulation in *A. flavus*. (**D**) Comparison between 500 µg/mL stem, leaf, and root CHL extract; α-lipoic acid 1 mM is used as a reference. Error bars refer to mean values of four replicates ± SD.

Time-dependent aflatoxin production was analyzed by time-course experiments, where the kinetic of toxin accumulation was “real time”, determined by starting from 65 to 146 h after inoculum in CCM medium. As previously reported [27] the AF concentration in the control cultures progressively increased for up to 85–90 h, maintaining, from here on, a ‘plateau’ value. AF production in the *A. flavus* cultures treated with a 500 μg/mL extract concentration showed a similar time course, but the maximum quantity of toxins produced varied consistently, with the extract-dependent inhibition rate observed in the end-point cultures. Stem extracts did not significantly differ from each other in terms of global inhibition level, blocking AF accumulation to 50% of the control value (Figure 3A). On the contrary, leaf and root extracts resulted in a variable range of toxin containment: The AF accumulation course, in cultures treated with leaf extracts, split from control at 72 h after inoculum, reaching a peak at 89 h (Figure 3B). A similar pattern was observed in root MET treated cultures, whereas root CHL and root EA avoided AF accumulation already before 65 h (Figure 3C).

Due to the variety of synergistic phenomena occurring in the cell, the antioxidant activity (and resulting biological effect) may rarely be calculated on the basis of chemical in vitro assays, mainly in the case of botanicals and phytocomplexes as those reported here. Therefore, the effect of different extracts on the redox balance could only in part be predicted. Additionally, the use of high concentrations of single constituents (when, actually, a plant extract is a complex mixture) may result in the in vitro system being exposed to an overstated and unrealistic concentration. However, a comparison with a standard molecule, owning well-documented antioxidant properties consistent with the biological effect in the object of this study, should be done. Lipoic acid is a well-known ROS scavenger, and its efficacy in preventing aflatoxin production in *A. flavus* was already reported [27]. At the highest dose considered here (500 µg/mL), CHL root extracts resulted in AF accumulation containment, which was comparable to that observed for 1 mM α-lipoic acid. Stem and leaf CHL extracts were less efficient inhibitors of AF biosynthesis, as compared to α-lipoic acid and root CHL extracts. It thus appears that CHL root extracts are a promising source of anti-aflatoxigenic molecules. On the other hand, reinforcing evidence shows that *A. flavus* aflatoxin-producing strains may be used as an in vivo model to test the antioxidant activity of new mixture/compounds.

### 2.4. Time Course of Extract Administration on Aflatoxin Accumulation

**Figure 4:**
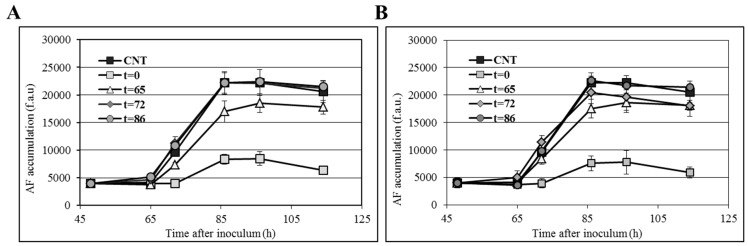
Effect of 500 µg/mL CHL (**A**) and EA (**B**) root extract over time on aflatoxin production. Extracts were added to conidia of *A. flavus* inoculated in CCM after 65, 72, and 86 h of incubation. Error bars refer to mean values of four replicates ± SD.

According to various authors, many plant extracts showing an inhibitory effect against aflatoxin accumulation at the early stage seemed to become almost ineffective after protracted incubation [56,57], suggesting that their biological activity might depend not only on phytochemical composition, but also on the chemical structure and related properties of single components. Additionally, evidence has been provided showing that the delivery time of an anti-oxidant compound during fungal growth may affect its inhibitory efficacy on aflatoxin biosynthesis and/or accumulation [40]. In Figure 4, the time course of *C. colocynthis* root extracts (CHL and EA) administration is reported. As a general observation, early administration (time 0; at the germination stage) of the relevant extract in the medium resulted in the highest inhibition of AF accumulation, whereas delaying the time of administration (from 65 h onwards) did not block mycotoxin biosynthesis but, in the best case (65 h), retarded aflatoxin accumulation.

As previously reported for lipoic acid [27], the efficacy of CHL and EA root extracts in preventing AF accumulation is limited to a short time interval (0–65 h) that precedes the burst of AF biosynthesis. It would be worth analyzing the metabolic and regulatory networks operating during this time window to uncover possible molecular targets for designing new and specific anti-aflatoxigenic compounds.

### 2.5. Conidia Production and Conidiophores Morphology

**Figure 5:**
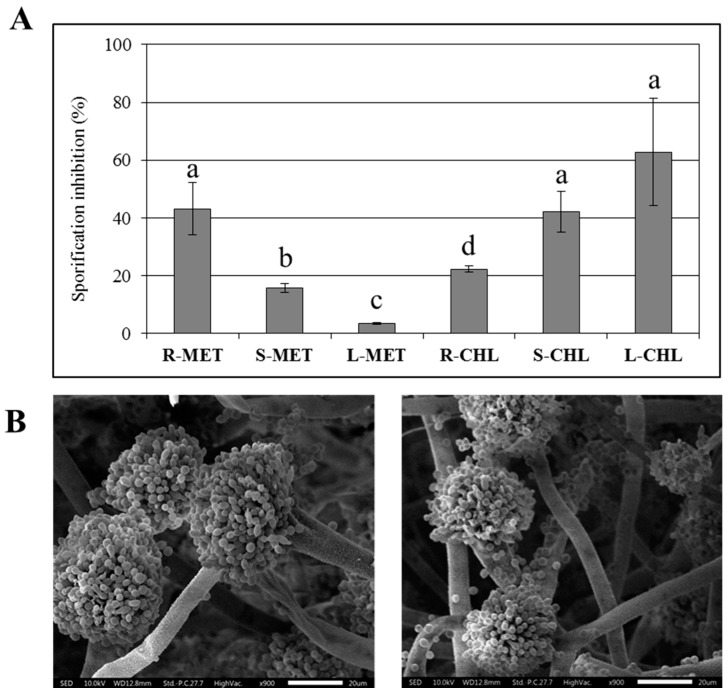
Effect of 500 µg/mL root, stem, leaf (R, S, L) MET and CHL extracts on conidia production (**A**) and conidiophores morphology (**B**) (left: Control; right: R-CHL treated cultures) in *A. flavus* 96-wells CCM cultures. Values are reported as inhibition percentages in respect to control; Error bars refer to mean values of four replicates ± S.D. Different letters over the bars indicate the differences that were statistically significant (*p* < 0.05).

In Aspergilla, vegetative reproduction and subsequent colonization of the surrounding environment rely on the differentiation of specialized structures (conidiophores) bearing vegetative spores (conidia), whose formation process characterizes the late phase of mycelium growth. We tested the effect of *C. colocynthis* CHL and MET stem, leaf, and root extracts on the production of conidia; as reported in Figure 5, a tissue and solvent (CHL and MET) dependent efficacy of the various extracts on lowering the number of conidia accumulated by treated mycelia was observed.

The overall pattern of tissue/solvent efficacy on conidia production was quite different with that observed for mycelium growth (Table 2) or hyphae elongation, and for AF accumulation (Figure 2). Interestingly, the scanning emission microscopy analysis (SEM) conducted to evaluate the conidiophore organization showed that no significant alteration of either the morphology or the general aspect of these reproductive structures occurred (an example is reported in Figure 5B), providing evidence that the relevant treatment affected the number of conidia or conidiophores.

## 3. Conclusions

Plants with significant pharmacological properties have often been found to be rich in polyphenols and other secondary metabolites that have been proven to possess high antioxidant potentials due to their activity as reducing agents, metal chelators, and free radical quenchers. In this sense, every bioactive able to interfere with the oxidative status of fungal cells, on which the mycotoxin metabolism relies, should be validated as a biocontrol agent in organic strategies aimed at reducing aflatoxin contamination in food and feed commodities. On the other hand, a search for new antifungal and antimycotoxigenic substances is of increasing interest, with the perspective of improving antifungal resistance and understanding the underlying mechanisms of these new drugs with a wide range of applications—from medical mycology to agricultural and food safety.

## 4. Materials and Methods

### 4.1. Plant Materials

*Citrullus colocynthis* L. Schrader plants were collected near Medenine (Tunisia), in the municipality of Sidi Makhlouf. The identification was performed according to the flora of Tunisia [58] and a voucher specimen (C.C-01.01) deposited in the biological laboratory of the Faculty of Pharmacy of Monastir.

### 4.2. Extraction Protocol

Fresh tissues (roots, stems, and leaves) were dried and powdered using a tissue blender. Different solvents, in ascending polarity (petroleum ether, chloroform, ethyl acetate, and methanol) were used for Soxhlet extraction to fractionate the soluble compounds from the plant material. The extraction was performed with dried powder (100 g) placed inside a thimble made by thick filter paper, loaded into the main chamber of the Soxhlet extractor, which consisted of an extracting tube, a glass balloon, and a condenser. The total extracting time was 6 h for each solvent, continuously refluxing over the sample at a temperature not exceeding the boiling point. The resulting extracts were evaporated at reduced pressure to obtain the crude extracts. The organic solvents used were 99% pure. Extracts were all ethanol resuspended for further analysis. All the chemicals were obtained from Sigma (St. Louis, MO, USA).

### 4.3. Determination of the Total Phenolic Contents

Phenolic compound concentration in the different extracts was determined by using the Folin–Ciocalteu’s phenol reagent, according to Singleton and Rossi [59], with some modifications. Briefly, 100 µL of the extract solution was mixed with 100 µL of Folin–Ciocalteu’s phenol reagent. After 3 min, 100 µL of saturated sodium carbonate solution was added to the mixture and adjusted to 1 mL with distilled water. The reaction was kept in the dark for 90 min, after which the absorbance was recorded at 720 nm. Gallic acid was used to design the standard curve. The contents of total phenolic are expressed as mg of gallic acid equivalents (GAE)/g of extract. Data were reported as means of three replicates ± S.D.

### 4.4. Determination of DPPH Radical Scavenging Activity

The ability to scavenge the DPPH-free radical was monitored according to a method first introduced by Blois (1958) and developed by Brand-Williams et al. [60]. Various concentrations of sample extracts (0.5 mL) were mixed with 0.5 mL of methanolic solution containing DPPH radicals (6 × 10^−5^ M). The mixture was shaken vigorously and left to stand in the dark until stable absorption values were obtained. The reduction of the DPPH radical was measured by continuously monitoring the decrease of absorption at 517 nm. The DPPH scavenging effect was calculated as a percentage of DPPH discoloration using the following equation: % scavenging effect = [(ADPPH × AS)/ADPPH] × 100, where AS is the absorbance of the solution when the sample extract has been added at a particular level, and ADPPH is the absorbance of the DPPH solution. Three experiments were performed in triplicate. The antiradical activity was expressed in terms of the amount of antioxidant necessary to decrease the initial DPPH absorbance by 50% (IC50). The IC50 value for each extract was determined graphically by plotting the percentage of DPPH scavenging as a function of extract concentration.

### 4.5. UPLC-DAD-ESI-MS/MS Analysis

For the chemical characterization, 60 mg of each extract were re-dissolved in methanol-distilled water (1:1 *v*/*v*) and filtered with a PTFE membrane. 5 µL were injected in a LC–DAD-MS/MS system, consisting of a Shimadzu Nexera UPLC system (Kyoto, Japan) coupled with a diode array detector (DAD), and a Shimadzu LCMS-8030 quadrupole mass spectrometer (Kyoto, Japan) equipped with a electrospray ionization source (ESI). Analytical separation was performed on a reversed-phase Waters Nova-Pak C18 column (4.9 × 250 mm, 4 µm) (Water Milford, MA, USA), operating at 30 °C. The mobile phase consisted of 1% aqueous formic acid (solvent A) and 1% formic acid in acetonitrile (solvent B). The elution gradient consisted of 3% B isocratic for 5 min, from 5 to 100% B linear for 30 min, 100% B isocratic for 7 min. The flow rate was 0.5 mL/min. The mass spectrometer operated in Negative Ion Scan and in Product Ion Scan mode, acquiring over a mass-range from *m*/*z* 50 to 1100 and using Argon as Collision Induced Dissociation (CID) gas at a pressure of 230 kPa. The interface voltage was set to −3.5 kV; desolvation line (DL) temperature was 250 °C and the heat block temperature was 400 °C.

Identification of the major secondary metabolites in the different extracts was carried out using their retention times, and both UV-VIS, MS and MS/MS spectra. Quantification of single compounds was performed by UPLC-DAD in triplicates through an external standard method, using stock solutions of the following compounds: Esculetin, p-coumaric acid, orientin, catechin, vitexin, (all from Sigma-Aldrich, Milan, Italy) and cucurbitacin I, cucurbitacin E, and apigenin-7-O-glucoside from Extrasynthese (Lyon, France). All solvents used for the analyses were purchased from Sigma-Aldrich (Milan, Italy).

### 4.6. Fungal Strains, Media and Culture Condition

*Aspergillus flavus* toxigenic strain Fri2 and atoxigenic strain TOϕ used were previously isolated from corn fields of the Po Valley [61]. Conidia suspensions were obtained from 10-day YES-agar [2% (*w*/*v*) yeast extract (Difco, Detroit, MI, USA), 5% (*w*/*v*) sucrose (Sigma, St Louis, MO, USA), 2% (*w*/*v*) agar (Difco)] cultures incubated at 28 °C; conidia concentration (quantified by OD_600_) and viability (>90%) were determined according to Degola et al. [42]. Coconut milk-derived medium (CCM) used for microplate assays was obtained as described in Degola et al. [27]: Briefly, 400 mL of commercial coconut cream was diluted to the final volume of 1.2 L with bidistilled water, sterilized by autoclaving (10 min, 120 °C), cooled at 4 °C overnight, and clarified by centrifugation (15 min at 3200× *g*). The residual floating material and the pellet were discarded, and the intermediate phase was then recovered and used as culture medium in the aflatoxin inhibition assays.

### 4.7. Aflatoxin Production Assay

The extracts’ effects on aflatoxin biosynthesis were assessed by the microplate fluorescence-based procedure described in Degola et al. [27]. Standard flat-bottom 96-well microplates (Sarstedt, Newton, NC, USA) were used. Suspensions of conidia were diluted to the appropriate concentrations and brought to the final concentration of 5 × 10^2^ conidia/well; cultures were set in a final volume of 200 µL/well of CCM medium. *C. colocynthis* organic extracts were ethanol resuspended and added to the culture medium. The plates were incubated in the dark under stationary conditions for up to 6 days at 25 °C; visual inspection of mycelium development and conidiation served as an indicator of the culture growth. Total aflatoxin accumulation was monitored by fluorescence emission determination; readings were performed directly from the wells bottom of the culture plate with a microplate reader (TECAN SpectraFluor Plus, Männedorf, Switzerland) using the following parameters: λ_ex_ = 360 nm; λ_em_ = 465 nm; manual gain = 83; lag time = 0 µs; number of flashes = 3; integration time = 200 µs). Inocula were performed in quadruplicate.

### 4.8. Aspergillus Flavus Growth

*A. flavus* radial growth was performed in YES-agar added with *C. colocynthis* extracts at 500 µg/mL final concentration: Three equidistant single spots (5 µL of a 10^7^ conidia/mL suspension each) of aflatoxigenic strain Fri2 were inoculated in Petri dishes (9 cm Ø), plates were incubated for 4 days at 25 °C, and the mycelium growth was evaluated daily by measuring colonies reverse along two orthogonal diameters. YES-agar plates supplemented with 0.5% EtOH (*v*/*v*) were used as control. Early mycelium development was assessed, recording changes in optical density of liquid cultures over time: In a 96 wells microtiter plate (Sarstedt, Newton, NC, USA), 1 × 10^4^ conidia were inoculated in a final volume of 200 µL of YES 5% liquid medium, added with 500 µg/mL organic extracts, and incubated at 28 °C. The optical density at 620 nm (OD_620_) was recorded for each well, using a microplate reader (RosysAnthos ht3; AnthosLabtec Instruments GmbH, Salzburg) without shaking. Experiments were performed in quadruplicate.

### 4.9. Conidiation Rate Assessment and Reproductive Structures Analysis

Conidia production was estimated for CHL and MET leaf, stem, and root extract treated cultures (500 µg/mL). From the eight replicates of each condition, four mycelia were collected from the CCM microplates used in the AF accumulation assay, and individually washed three times in a 0.1% (*v*/*v*) Tween20^®^ aqueous solution by vortexing 1 min. The spore suspensions were then washed three times with a 80% (*v*/*v*) ethanol solution and conidia concentration was then determined with a Burker chamber. The remaining four CHL and MET leaf, stem, and root extract treated cultures from AF accumulation plates were observed using a Scanning Electron Microscope (SEM) JEOL IT 300, in high vacuum mode. Samples were fixed for 4 h in 3% (*v*/*v*) glutaraldehyde, acetone dehydrated (from 30% to 100% water/acetone solutions), critical point dried, and coated with a thin layer of gold by means of a Sputter Coater “Agar”. Observations were conducted at an acceleration voltage of 10.0 kV and at a 450× magnification.

### 4.10. Statistical Analysis

The data were analyzed using the statistical and graphical function of PASW Statistics (SPSS Inc., Chicago, IL, USA). Differences were assessed using analysis of variance (ANOVA), followed by Dunnet-t post hoc test.

## Figures and Tables

**Table 1:** UPLC-DAD-MS/MS characterization and quantification (µmol g^−1^ D.W.) of main secondary metabolites present in the different extracts of *C. colocynthis*. Data are means ± S.D. (*n* = 3). * nd = not detectable.

						Ethyl Acetate			Methanol			Chloroform		
n°	Name	MW	[M − H]^−^, *m*/*z*	MS/MS, *m*/*z*	λmax, nm	Leaf	Stem	Root	Leaf	Stem	Root	Leaf	Stem	Root
**1**	Esculetin	178	177	133, 105, 89	330	40.6 ± 4.91	10.5 ± 1.08	17.7 ± 2.03	n.d.	n.d.	n.d.	n.d.	n.d.	n.d.
**2**	p-Coumaric acid	164	163	147, 119	225, 310	20.7 ± 1.24	6.55 ± 0.45	1.67 ± 0.09	1.59 ± 0.12	1.75 ± 0.17	n.d.	n.d.	n.d.	n.d.
**3**	Orientin	448	447	327, 357, 285	350, 269	n.d.	n.d.	0.28 ± 0.01	140.3 ± 20.87	7.30 ± 0.97	1.20 ± 0.18	n.d.	n.d.	n.d.
**4**	Apigenin-hexoside	432	431	311, 269, 211, 159	267, 337	1.59 ± 0.23	0.41 ± 0.05	n.d.	n.d.	n.d.	n.d.	n.d.	n.d.	n.d.
**5**	trans p-Coumaric acid 4-*O*-malate	280	279	147, 119	225, 310	0.79 ± 0.09	1.89 ± 0.31	n.d.	2.837 ± 0.21	5.44 ± 0.61	n.d.	n.d.	n.d.	n.d.
**6**	Vitexin	432	431	311, 341, 269	267, 337	3.86 ± 0.04	n.d.	n.d.	7.69 ± 0.68	4.66 ± 0.59	n.d.	n.d.	n.d.	n.d.
**7**	Apigenin -D-glucopyranosyl-8-apiofuranoside	564	563	311, 269	267, 337	59.6 ± 0.73	13.10 ± 1.44	n.d.	529.1 ± 30.17	108.5 ± 11.39	n.d.	n.d.	n.d.	n.d.
**8**	Apigenin derivative (isomer 1)	548	547	311	267, 337	13.5 ± 1.70	3.16 ± 0.29	0.05 ± 0.00	4.6 ± 0.42	0.31 ± 0.00	0.7	n.d.	n.d.	n.d.
**9**	Apigenin derivative (isomer 2)	548	547	311	267, 337	47.7 ± 3.99	14.3 ± 1.74	0.28 ± 0.01	13.6 ± 0.15	8.09 ± 0.06	1.77	n.d.	n.d.	n.d.
**10**	caffeoyl malic Flavone	578	577	179	340, 270	3.08 ± 0.29	1.19 ± 0.02	n.d.	n.d.	n.d.	n.d.	n.d.	n.d.	n.d.
**11**	coumaric Flavone derivative	584	583	285, 147	348, 310	4.88 ± 0.51	5.06 ± 0.61	0.08 ± 0.00	55.1 ± 6.48	6.68 ± 0.52	1.34 ± 0.14	n.d.	n.d.	n.d.
**12**	Apigenin derivative	752	751	311	267, 337	n.d.	63.6 ± 5.41	24.9 ± 1.84	12.8 ± 2.54	n.d.	n.d.	n.d.	n.d.	n.d.
**13**	Cucurbitacin E	556	555	n.d.	229	54.2 ± 6.0	57.9 ± 6.9	13. 6 ± 0.98	n.d.	6.84 ± 0.54	2.48 ± 0.54	44.5 ± 5.41	55.9 ± 7.82	14.5 ± 2.54
**14**	Cucurbitacin I	514	513	n.d.	229	99.8 ± 8.21	5.81 ± 0.42	27.6 ± 3.41	n.d.	n.d.	n.d.	212.6 ± 32.77	220.2 ± 19.9	41.3 ± 6.77
**15**	acetyl Cucurbitacin E	760	759	n.d.	229	9.31 ± 0.57	n.d.	3.59 ± 0.08	n.d.	n.d.	n.d.	n.d.	n.d.	n.d.
**16**	coumaroyl acetyl Cucurbitacin I	864	863	n.d.	229	7.13 ± 0.44	n.d.	0.99 ± 0.00	n.d.	n.d.	n.d.	36.6 ± 4.09	n.d.	n.d.
**17**	Apigenin-dihexoside	594	593	311	267, 337	n.d.	n.d.	n.d.	15.4 ± 2.01	18.1 ± 2.55	n.d.	n.d.	n.d.	n.d.
**18**	Epicatechin gallate	442	441	289	280	n.d.	n.d.	n.d.	16.8 ± 2.11	10.8 ± 0.98	n.d.	n.d.	n.d.	n.d.
**19**	Colocynthoside B	806	805	n.d.	230	n.d.	n.d.	n.d.	n.d.	n.d.	n.d.	25.6 ± 3.29	3.89 ± 0.45	6.69 ± 0.71

**Table 2:** Effect of 500 µg/mL *C. colocynthis* extracts on *A. flavus* radial growth. Radial increment is expressed as the mean of daily radial increase of colonies radius (cm/d) ± S.D. Same letters indicate absence of statistically significant differences (*p* < 0.05).

	CHL	EA	MET	CNT
**Root**	0.45 ± 0.13 ^a^	0.43 ± 0.06 ^a^	0.43 ± 0.09 ^a^	0.47 ± 0.08 ^a^
**Stem**	0.45 ± 0.11 ^a^	0.39 ± 0.15 ^a^	0.44 ± 0.12 ^a^	0.47 ± 0.08 ^a^
**Leaf**	0.44 ±0.13 ^a^	0.46 ± 0.08 ^a^	0.43 ± 0.10 ^a^	0.47 ± 0.08 ^a^
